# Supplementary material for: A study of soil seed banks across one complete chronosequence of secondary succession in a karst landscape
Source: PeerJ. 2020 Oct 19;8:e10226. doi: 10.7717/peerj.10226 (PMC7580579; doi:10.7717/peerj.10226)
Supplement: Supplemental Information 2 — The important value of each species in eight succession stages. [file peerj-08-10226-s002.docx]

Supplemental Information I Plant species and their important values in different succession in Guiyang karst landscape

| Name of species | PEBF | SEBF | SF | TVSF | SGC-II | SGC-Ⅰ | GC-Ⅱ | GC-Ⅰ |  |
| --- | --- | --- | --- | --- | --- | --- | --- | --- | --- |
| **Woody plants** |  |  |  |  |  |  |  |  |  |
| *Carpinus pubescens* | 20.61 | 1.35 |  |  |  |  |  |  |  |
| *Platycarya strobilacea Sieb.et Zucc* | 11.30 | 1.44 |  |  |  | 1.37 |  |  |  |
| *Itea yunnanensis* | 8.59 | 11.42 | 2.3 | 4.93 | 2.04 | 1.34 |  |  |  |
| *Myrsine africana* | 4.62 | 4.34 | 4.03 | 3.74 | 2.16 |  |  |  |  |
| *Rhamnus heterophylla* | 3.97 | 2.19 | 1.27 | 15.16 | 13.97 | 3.69 |  |  |  |
| *Viburnum utile* | 3.20 | 5.6 |  | 14.27 | 3.02 | 21.25 |  |  |  |
| *Zanthoxylum scandens* Bl. | 2.46 |  |  |  |  |  |  |  |  |
| *Toddalia asiatica* | 2.13 |  | 1.96 | 3.38 | 1.91 |  |  |  |  |
| *Pyracantha fortuneana* | 2.03 | 7.35 | 1.98 | 10.69 | 18.26 | 20.89 |  |  |  |
| *Ulmus pumila* L. | 2.02 |  |  |  |  |  |  |  |  |
| *Smilax* *china* L. | 2.00 |  | 1.41 |  |  |  |  |  |  |
| *Zanthoxylum planispinum* Sieb.et Zucc*.* | 1.95 | 2.29 | 1.42 | 8.93 | 7.4 |  |  |  |  |
| *Ligustrum lucidum* | 1.79 | 2.67 |  |  |  |  |  |  |  |
| *Rubus biflorus* | 1.67 | 1.25 | 4.5 | 5.09 | 3.56 | 1.63 |  |  |  |
| *Lindera communis* | 1.65 | 1.57 | 3.65 |  |  | 9.22 |  |  |  |
| *Ilex corallina var. aberrans* | 1.56 |  |  |  |  |  |  |  |  |
| *Corylus heterophylla* var. sutchuenensis | 1.55 |  |  |  |  |  |  |  |  |
| *Serissa japonica* | 1.49 |  |  |  |  |  |  |  |  |
| *Ilex chinensis* | 1.47 |  |  |  |  |  |  |  |  |
| *Cyclobalanopsis glauca* | 1.40 |  |  |  |  |  |  |  |  |
| *Daphne acutiloba* | 1.25 |  |  |  |  |  |  |  |  |
| *Dalbergia hancei* | 1.24 |  |  |  |  |  |  |  |  |
| *Photinia davidsoniae* | 1.18 |  |  |  |  |  |  |  |  |
| *Albizia kalkora* | 1.17 |  |  |  |  |  |  |  |  |
| *Robinia pseudoacacia* | 1.10 |  |  |  |  |  |  |  |  |
| *Celtis biondii* | 1.10 |  |  |  |  | 1.08 |  |  |  |
| *Evodia rutaecarpa* | 1.09 | 1.2 | 1.12 |  | 1.53 |  |  |  |  |
| *Cercis chinensis* | 1.08 |  |  |  |  |  |  |  |  |
| *Vaccinium bracteatum* | 1.03 |  |  |  |  |  |  |  |  |
| *Rosa cymosa* |  | 7.73 | 7.33 |  | 7.56 | 10.26 |  |  |  |
| *Quercus fabri* |  | 7.14 | 4.6 |  |  |  |  |  |  |
| *Platycarya longipes Wu* |  | 6.27 |  |  |  |  |  |  |  |
| *Rhus punjabensis* var. sinica |  | 6.21 | 1.33 |  | 2.01 | 1.05 |  |  |  |
| *Cotinus coggygria* var. glaucophylla |  | 4.81 | 20.97 |  |  | 3.33 |  |  |  |
| *Populus adenopoda* |  | 3.53 | 1.49 |  |  |  |  |  |  |
| *Rhus chinensis* |  | 2.17 |  |  |  |  |  |  |  |
| *Coriaria nepalensis* |  | 1.92 | 1.92 |  | 1.23 | 13.82 |  |  |  |
| *Loropetalum chinense* |  | 1.45 | 1.94 |  |  |  |  |  |  |
| *Aphananthe aspera* |  | 1.23 |  |  |  |  |  |  |  |
| *Zanthoxylum esquirolii* |  | 1.21 |  |  |  |  |  |  |  |
| *Rhododendron rivulare* |  | 1.17 |  |  |  |  |  |  |  |
| *Millettia dielsiana* |  | 1.14 | 1.86 | 1.24 |  |  |  |  |  |
| *Rhamnus leptophylla* |  | 1.09 | 1.92 |  |  |  |  |  |  |
| *Elaeagnus pungens* |  | 1.06 | 1.6 |  |  |  |  |  |  |
| *Flueggea virosa* |  |  | 4.49 |  |  |  |  |  |  |
| *Pouzolzia sanguinea* |  |  | 2.64 |  |  |  |  |  |  |
| *Prinsepia utilis* |  |  | 2.4 |  |  |  |  |  |  |
| *Rubus parvifolius* |  |  | 2.06 | 1.9 | 1.88 |  |  |  |  |
| *Rubus setchuenensis* |  |  | 1.64 |  | 1.42 |  |  |  |  |
| *Ampelopsis sinica* |  |  | 1.6 |  |  |  |  |  |  |
| *Broussonetia papyrifera* |  |  | 1.49 |  | 1.01 |  |  |  |  |
| *Viburnum foetidum var. ceanothoides* |  |  | 1.42 | 1.06 | 1.08 | 1.24 |  |  |  |
| *Nandina domestica* |  |  | 1.34 |  |  |  |  |  |  |
| *Ligustrum sinense* |  |  | 1.22 |  |  | 1.03 |  |  |  |
| *hypericum patulum* |  |  |  | 7.2 | 14.45 |  |  |  |  |
| *Indigofera pseudotinctoria* |  |  |  | 1.69 |  | 1.02 |  |  |  |
| *Cotoneaster horizontalis* var. perpusillus |  |  |  | 1.13 |  |  |  |  |  |
| *Ficus tikoua* |  |  |  |  | 8.84 |  |  |  |  |
| *Litsea rubescens* |  |  |  |  | 1.43 |  |  |  |  |
| *Rubus pinfaensis* |  |  |  |  |  | 1.83 |  |  |  |
| *Sophora xanthantha* |  |  |  |  |  | 1.77 |  |  |  |
| *Rubus faberi* |  |  |  |  |  | 1.53 |  |  |  |
| *Clerodendrum bungei* |  |  |  |  |  | 1.15 |  |  |  |
| **Herb plants** |  |  |  |  |  |  |  |  |  |
| *Digitaria sanguinalis* |  |  |  | 4.51 | 3.44 | 27.16 | 22.14 | 18.13 |  |
| *Artemisia roxburghiana* |  |  |  | 2.68 | 2.16 |  | 12.2 |  |  |
| *Trifolium repens* |  |  |  | 1.47 | 3.78 | 15.74 | 10.56 | 19.31 |  |
| *Heteropogon contortus* |  |  | 4.09 |  | 5.24 |  | 6.43 |  |  |
| *Arthraxon hispidus* | 5.13 | 12.58 | 1.83 | 2.65 | 11.34 | 26.1 | 4.43 | 3.52 |  |
| *Erigeron annuus* |  |  | 1.97 |  | 4.06 | 1.79 | 3.56 |  |  |
| *Centella asiatica* |  |  |  | 3.14 | 2.73 |  | 3.44 | 2.22 |  |
| *Clinopodium chinense* |  |  | 2.37 | 1.33 | 3.09 | 1.27 | 3.01 |  |  |
| *Stellaria media* | 1.63 | 6.09 | 6.12 | 1.65 | 2.21 | 2.07 | 2.79 | 1.42 |  |
| *Setaria viridis* | 4.19 | 3.33 | 3.46 |  | 2.44 | 3.41 | 2.6 |  |  |
| *Dendranthema indicum* |  |  |  | 3.1 | 1.56 |  | 2.58 |  |  |
| *Bergenia purpurascens* |  |  |  |  |  |  | 2.25 |  |  |
| *Kummerowia striata* |  |  |  | 2.06 | 1.69 |  | 1.54 | 1.64 |  |
| *Duchesnea indica* |  | 3.46 |  | 3.11 |  | 4.3 | 2.29 |  |  |
| *Veronica didyma* |  |  |  |  | 1.47 |  | 1.71 |  |  |
| *Arthraxon lanceolatus* |  | 8.93 |  | 17.73 |  |  | 1.42 |  |  |
| *Brachiaria eruciformis* | 30.42 |  |  |  |  |  | 1.69 | 1.94 |  |
| *Adenostemma lavenia* |  |  |  |  |  |  | 1.61 |  |  |
| *Polygonum nepalense* |  |  |  |  |  |  | 1.74 |  |  |
| *Solanum nigrum* |  |  |  |  |  |  | 1.53 |  |  |
| *Acalypha australis* |  |  |  | 1.24 |  |  | 1.69 |  |  |
| *Anaphalis bulleyana* |  |  |  |  |  |  | 1.92 |  |  |
| *Keiskea elsholtzioides* |  | 7.21 |  |  |  |  | 1.24 |  |  |
| *Digitaria ciliaris* |  |  |  |  |  |  | 1.06 | 1.99 |  |
| *Anaphalis margaritacea* |  |  |  |  |  | 1.54 | 1.2 | 2.29 |  |
| *Hydrocotyle sibthorpioides* |  |  |  | 2.84 | 3.63 |  |  | 22.11 |  |
| *Plantago asiatica* |  |  |  | 4.65 | 3.14 | 1.88 |  | 5.14 |  |
| *Taraxacum mongolicum* |  |  |  |  |  |  |  | 4.08 |  |
| *Carex lanceolata* Boott var*.* | 8.71 | 6.15 | 23.1 | 14.31 | 4.21 | 1.32 |  |  |  |
| *Youngia heterophylla* | 2.1 |  | 2.14 | 3.41 | 2.37 |  |  | 2.01 |  |
| *Oxalis corniculata* |  |  |  | 1.21 | 2.83 |  |  | 2.01 |  |
| *Cyrtococcum patens* |  |  |  |  |  | 1.61 |  | 2.45 |  |
| *Digitaria ischaemum* |  |  |  |  |  |  |  | 2.38 |  |
| *Carex capilliformis* |  |  |  |  |  |  |  | 1.19 |  |
| *Achyranthes bidentata* |  |  |  | 1.98 | 1.15 |  | 1.65 | 1.76 |  |
| *Stellaria chinensis* |  |  |  |  | 2.28 |  |  | 1.28 |  |
| *Imperata cylindrica* |  | 4.43 | 4.61 | 6.71 | 20.14 | 4.1 |  |  |  |
| *Verbena officinalis* |  |  |  |  |  | 2.61 |  |  |  |
| *Anemone rivularis* |  |  |  |  |  | 1.07 |  |  |  |
| *Aster menelii Levl.* |  |  |  |  |  | 1.03 |  |  |  |
| *Thalictrum aquilegifolium* |  | 9.68 | 6.13 |  | 3.96 |  |  |  |  |
| *Pteris cretica* | 9.16 | 8.34 | 7.77 | 9.73 | 2.04 |  |  |  |  |
| *Eulalia speciosa* |  |  |  | 1.21 | 2.81 |  |  |  |  |
| *Trisetum bifidum* |  |  |  | 10.53 | 2.67 |  |  |  |  |
| *Vicia unijuga* |  |  |  |  | 2.11 |  |  |  |  |
| *Erianthus rufipilus* |  |  | 4.62 | 6.78 |  |  |  |  |  |
| *Rubia cordifolia* |  |  | 2.68 | 2.19 |  |  |  |  |  |
| *Boehmeria macrophylla Hornem* | 2.82 |  | 3.14 | 2.81 |  |  |  |  |  |
| *Bidens pilosa* |  | 6.78 | 4.42 |  |  |  |  |  |  |
| *Viola verecunda* |  |  | 2.23 |  |  |  |  |  |  |
| *Conyza japonica* |  |  | 1.44 |  |  |  |  |  |  |
| *Aster ageratoides* Turcz. |  | 6.39 |  |  |  |  |  |  |  |
| *Kalimeris indica* |  | 3.46 |  |  |  |  |  |  |  |
| *Lespedeza cuneata* |  | 3.51 |  |  |  |  |  |  |  |
| *Ixeris polycephala* Cass |  | 4.14 |  |  |  |  |  |  |  |
| *Senecio scandens* | 6.53 | 2.17 | 3.16 | 2.34 |  |  |  |  |  |
| *Lilium brownii* |  | 2.89 |  |  |  |  |  |  |  |
| *Epimedium brevicornu* Maxim | 17.23 |  |  |  |  |  |  |  |  |
| *Rabdosia amethystoides* | 6.64 |  |  |  |  |  |  |  |  |
| *Pyrrosia lingua* | 5.44 |  |  |  |  |  |  |  |  |
