# Supplementary material for: A study of soil seed banks across one complete chronosequence of secondary succession in a karst landscape
Source: PeerJ. 2020 Oct 19;8:e10226. doi: 10.7717/peerj.10226 (PMC7580579; doi:10.7717/peerj.10226)
Supplement: Supplemental Information 3 — The names of all plant species tested from soil samples, and corresponding seed numbers before and after germination in eight succession stages. [file peerj-08-10226-s003.docx]

Supplemental information II Seed density of all recorded species in different successions (seedling)

| *The species name* | GC-I | |  | GC-Ⅱ | |  | SGC-Ⅰ | | | |  | | SGC-Ⅱ | | | |  | | VTSF | | | |  | | SF | | | |  | | SEBF | | | |  | | PEBF | | | | | Total | |  |
| --- | --- | --- | --- | --- | --- | --- | --- | --- | --- | --- | --- | --- | --- | --- | --- | --- | --- | --- | --- | --- | --- | --- | --- | --- | --- | --- | --- | --- | --- | --- | --- | --- | --- | --- | --- | --- | --- | --- | --- | --- | --- | --- | --- | --- |
|  | B | A | | B | A | | B | | A | | | | B | | A | | | | B | | A | | | | B | | A | | | | B | | A | | | | B | | | A | |  | |  |
| **HERB** |  |  |  |  |  | |  |  | |  | |  | |  | |  | |  | |  | |  | |  | |  | |  | |  | |  | |  | |  | |  | | |  | |  | |
| *Carex lanceolata Boott var.* |  | 9 |  | 38 | 32 | |  | 54 | | 29 | |  | | 61 | | 33 | |  | | 101 | | 43 | |  | | 61 | | 33 | |  | | 106 | | 72 | |  | | 100 | | | 81 | | **853** | |
| *Digitaria sanguinalis* | 83 | 50 |  | 101 | 35 | |  | 59 | | 28 | |  | | 80 | | 43 | |  | | 53 | |  | |  | | 37 | | 24 | |  | | 40 | | 36 | |  | | | 33 | | 26 | | **728** | |
| *Arthraxon hispidus* | 66 | 17 |  | 66 | 27 | |  | 65 | | 33 | |  | | 68 | | 11 | |  | | 57 | | 12 | |  | | 40 | | 18 | |  | | 62 | | 33 | |  | | | 46 | | 21 | | **642** | |
| *Setaria viridis* | 39 |  |  | 63 |  | |  | 60 | | 18 | |  | | 52 | | 7 | |  | | 48 | |  | |  | | 32 | |  | |  | | 64 | | 11 | |  | | | 39 | |  | | **433** | |
| *Oxalis corniculata* | 12 | 34 |  | 36 | 26 | |  | 36 | | 20 | |  | | 21 | | 29 | |  | | 8 | | 24 | |  | | 6 | | 23 | |  | | 61 | | 50 | |  | | |  | | 42 | | **428** | |
| *Centella asiatica* | 16 | 13 |  | 54 | 13 | |  | 37 | | 15 | |  | |  | | 27 | |  | |  | | 20 | |  | | 19 | | 18 | |  | | 67 | | 31 | |  | | | 26 | | 63 | | **419** | |
| *Brachiaria eruciformis* | 31 | 6 |  | 66 | 22 | |  | 24 | | 11 | |  | | 35 | |  | |  | | 9 | | 2 | |  | | 20 | | 16 | |  | | 23 | | 32 | |  | | | 22 | | 56 | | **375** | |
| *Arthraxon lanceolatus* |  | 17 |  | 38 | 27 | |  | 31 | | 7 | |  | | 38 | | 12 | |  | | 27 | | 13 | |  | | 22 | |  | |  | | 38 | | 40 | |  | | | 38 | | 16 | | **364** | |
| *Stellaria media* | 14 | 23 |  | 42 | 23 | |  | 16 | | 24 | |  | | 49 | | 14 | |  | | 27 | | 10 | |  | | 11 | | 20 | |  | | 25 | |  | |  | | | 23 | | 16 | | **337** | |
| *Achyranthes bidentata* | 34 |  |  | 16 | 6 | |  | 32 | | 3 | |  | | 41 | | 2 | |  | | 22 | |  | |  | | 21 | | 8 | |  | | 39 | | 8 | |  | | | 32 | |  | | **264** | |
| *Conyza japonica* |  |  |  | 35 |  | |  | 37 | | 6 | |  | | 15 | | 12 | |  | | 44 | | 10 | |  | | 23 | | 6 | |  | | 18 | | 14 | |  | | |  | | 8 | | **228** | |
| *Carex capilliformis* | 25 |  |  |  |  | |  | 16 | | 9 | |  | |  | | 14 | |  | | 18 | | 9 | |  | | 23 | | 7 | |  | | 8 | | 27 | |  | | | 23 | | 14 | | **193** | |
| *Digitaria ciliaris* |  | 17 |  |  |  | |  | 35 | |  | |  | | 16 | |  | |  | | 30 | | 11 | |  | | 10 | | 5 | |  | |  | |  | |  | | |  | |  | | **124** | |
| *Trisetum bifidum* |  |  |  |  |  | |  |  | |  | |  | | 24 | |  | |  | | 18 | | 6 | |  | | 23 | |  | |  | | 34 | |  | |  | | | 16 | |  | | **121** | |
| *Hydrocotyle sibthorpioides* | 52 | 15 |  | 16 | 5 | |  |  | |  | |  | |  | | 17 | |  | |  | |  | |  | |  | |  | |  | |  | |  | |  | | |  | |  | | **105** | |
| *Acalypha australis* |  |  |  |  | 7 | |  | 17 | |  | |  | |  | |  | |  | | 23 | | 7 | |  | | 8 | |  | |  | | 36 | | 2 | |  | | |  | |  | | **100** | |
| *Heteropogon contortus* | 21 | 22 |  | 37 | 18 | |  |  | |  | |  | |  | |  | |  | |  | |  | |  | |  | |  | |  | |  | |  | |  | | |  | |  | | 98 | |
| *Bidens pilosa* |  |  |  |  |  | |  |  | |  | |  | | 18 | |  | |  | | 3 | | 5 | |  | | 4 | | 12 | |  | | 24 | | 7 | |  | | | 15 | | 9 | | 97 | |
| *Rabdosia amethystoides* |  |  |  |  | 3 | |  |  | | 2 | |  | | 15 | | 3 | |  | | 17 | |  | |  | | 14 | | 4 | |  | | 13 | | 6 | |  | | | 13 | |  | | 90 | |
| *Trifolium repens* | 31 | 11 |  | 22 | 21 | |  |  | |  | |  | |  | |  | |  | |  | |  | |  | |  | |  | |  | |  | |  | |  | | |  | |  | | 85 | |
| *Kummerowia striata* | 4 |  |  |  |  | |  |  | |  | |  | |  | |  | |  | | 12 | |  | |  | |  | |  | |  | | 41 | |  | |  | | |  | | 17 | | 74 | |
| *Erigeron annuus* |  | 8 |  | 14 | 10 | |  |  | | 12 | |  | | 7 | | 9 | |  | |  | | 5 | |  | |  | |  | |  | |  | | 7 | |  | | |  | |  | | 72 | |
| *Artemisia roxburghiana* |  | 1 |  | 25 |  | |  | 9 | |  | |  | | 6 | | 3 | |  | |  | | 6 | |  | | 11 | |  | |  | |  | |  | |  | | |  | | 11 | | 72 | |
| *Youngia heterophylla* | | 12 |  | 9 |  | |  |  | | 8 | |  | |  | | 4 | |  | | 10 | | 4 | |  | |  | | 7 | |  | |  | | 6 | |  | | |  | | 10 | | 70 | |
| *Eulalia speciosa* |  |  |  |  |  | |  |  | | 4 | |  | | 18 | |  | |  | | 8 | |  | |  | | 17 | |  | |  | | 11 | |  | |  | | | 10 | |  | | 68 | |
| *Kalimeris indica* |  |  |  |  | 3 | |  | 8 | | 18 | |  | | 5 | | 5 | |  | |  | |  | |  | | 9 | | 8 | |  | |  | | 7 | |  | | |  | |  | | 63 | |
| *Senecio scandens* |  |  |  |  |  | |  |  | |  | |  | |  | |  | |  | |  | |  | |  | | 4 | | 6 | |  | | 21 | | 10 | |  | | | 13 | | 9 | | 63 | |
| *Euphorbia hirta* |  |  |  |  |  | |  |  | | 3 | |  | | 19 | | 5 | |  | | 4 | | 1 | |  | | 11 | |  | |  | | 17 | |  | |  | | |  | |  | | 60 | |
| *Potentilla chinensis Ser.* |  |  |  |  |  | |  | 7 | | 3 | |  | | 5 | | 4 | |  | |  | |  | |  | |  | | 3 | |  | | 18 | | 8 | |  | | |  | | 4 | | 52 | |
| *Cyrtococcum patens* | 20 | 5 |  |  | 3 | |  | 21 | |  | |  | |  | |  | |  | |  | |  | |  | |  | | 2 | |  | |  | |  | |  | | |  | |  | | 51 | |
| *Carex cruciata Wahlenb* |  |  |  |  |  | |  |  | |  | |  | |  | |  | |  | |  | | 9 | |  | |  | |  | |  | | 38 | |  | |  | | | 4 | |  | | 51 | |
| *Eupatorium japonicum Thunb* |  |  |  |  |  | |  |  | | 16 | |  | | 12 | |  | |  | |  | |  | |  | | 18 | |  | |  | |  | |  | |  | | |  | |  | | 46 | |
| *Clinopodium chinense* | 13 |  |  | 8 |  | |  | 3 | |  | |  | |  | |  | |  | | 6 | |  | |  | |  | | 4 | |  | | 5 | |  | |  | | |  | |  | | 39 | |
| *Stellaria chinensis* |  | 2 |  | 11 |  | |  |  | |  | |  | |  | | 2 | |  | |  | | 4 | |  | |  | | 8 | |  | |  | | 11 | |  | | |  | |  | | 38 | |
| *Plantago asiatica* | 13 | 14 |  | 5 |  | |  |  | | 1 | |  | |  | | 4 | |  | |  | |  | |  | |  | |  | |  | |  | |  | |  | | |  | |  | | 37 | |
| *Rubia cordifolia* |  |  |  |  |  | |  |  | |  | |  | |  | |  | |  | |  | |  | |  | | 4 | | 3 | |  | |  | | 7 | |  | | | 8 | | 12 | | 34 | |
| *Mazus japonicus(Thunb.)O.Kuntze* |  | 4 |  | 4 |  | |  |  | | 3 | |  | |  | |  | |  | | 7 | |  | |  | | 14 | |  | |  | |  | |  | |  | | |  | |  | | 32 | |
| *Verbena officinalis L.* | 4 |  |  |  | 8 | |  |  | | 6 | |  | | 4 | |  | |  | |  | |  | |  | |  | | 9 | |  | |  | |  | |  | | |  | |  | | 31 | |
| *Anemone hupehensis* | |  |  |  |  | |  |  | |  | |  | | 5 | | 6 | |  | | 10 | | 5 | |  | |  | | 4 | |  | |  | |  | |  | | |  | |  | | 30 | |
| *Duchesnea indica* |  |  |  | 7 |  | |  | 3 | |  | |  | |  | |  | |  | | 2 | | 3 | |  | |  | |  | |  | |  | | 6 | |  | | |  | | 8 | | 29 | |
| *Dendranthema indicum* |  |  |  | 8 | 10 | |  |  | |  | |  | |  | |  | |  | |  | | 4 | |  | | 7 | |  | |  | |  | |  | |  | | |  | |  | | 29 | |
| *Urena lobata L.var.scabriuscula (DC.）Walp.* |  |  |  |  |  | |  |  | |  | |  | |  | | 15 | |  | |  | | 3 | |  | | 8 | |  | |  | |  | |  | |  | | | 3 | |  | | 29 | |
| *Hypericum erectum Thunb.ex Murr.* |  |  |  |  |  | |  |  | |  | |  | |  | |  | |  | | 5 | |  | |  | | 2 | |  | |  | | 4 | | 5 | |  | | | 6 | | 4 | | 26 | |
| *Anaphalis margaritacea* |  | 8 |  |  | 5 | |  | 9 | |  | |  | |  | |  | |  | |  | |  | |  | |  | |  | |  | |  | |  | |  | | |  | |  | | 22 | |
| *Viola verecunda* |  |  |  |  |  | |  | 4 | |  | |  | | 14 | |  | |  | |  | |  | |  | |  | |  | |  | |  | |  | |  | | | 3 | |  | | 21 | |
| *Veronica didyma* |  | 6 |  |  | 2 | |  | 11 | |  | |  | |  | |  | |  | |  | |  | |  | |  | |  | |  | |  | |  | |  | | |  | |  | | 19 | |
| *Amaranthus lividus* |  |  |  |  |  | |  |  | |  | |  | | 5 | |  | |  | |  | |  | |  | | 6 | |  | |  | | 6 | |  | |  | | |  | |  | | 17 | |
| *Viola yedoensis Makino* |  | 7 |  |  |  | |  |  | |  | |  | |  | | 9 | |  | |  | |  | |  | |  | |  | |  | |  | |  | |  | | |  | |  | | 16 | |
| *Pilea notata C. H. Wright* | |  |  |  |  | |  |  | |  | |  | |  | |  | |  | |  | |  | |  | |  | |  | |  | |  | | 5 | |  | | | 5 | | 5 | | 15 | |
| *Taraxacum mongolicum* | 9 |  |  |  | 3 | |  |  | |  | |  | |  | |  | |  | |  | |  | |  | |  | |  | |  | |  | |  | |  | | |  | |  | | 12 | |
| *Clinopodium megalanthum (Diels) C. Y. Wu et Hsuan ex H. W. Li* |  |  |  |  |  | |  | 6 | |  | |  | | 6 | |  | |  | |  | |  | |  | |  | |  | |  | |  | |  | |  | | |  | |  | | 12 | |
| *Polygonum foliosum* |  |  |  |  | 1 | |  |  | |  | |  | |  | |  | |  | | 3 | |  | |  | |  | | 4 | |  | | 4 | |  | |  | | |  | |  | | 12 | |
| *Adenostemma lavenia* |  |  |  | 8 |  | |  |  | |  | |  | |  | | 3 | |  | |  | |  | |  | |  | |  | |  | |  | |  | |  | | |  | |  | | 11 | |
| *Dioscorea opposita Thunb.* |  |  |  |  |  | |  |  | |  | |  | |  | |  | |  | | 3 | |  | |  | |  | |  | |  | | 8 | |  | |  | | |  | |  | | 11 | |
| *P. nepalense Meisn. nepalense Meisn.* |  |  |  | 10 |  | |  |  | |  | |  | |  | |  | |  | |  | |  | |  | |  | |  | |  | |  | |  | |  | | |  | |  | | 10 | |
| *Solanum nigrum L.* |  |  |  |  |  | |  |  | |  | |  | | 10 | |  | |  | |  | |  | |  | |  | |  | |  | |  | |  | |  | | |  | |  | | 10 | |
| *Eupatorium odoratum L.* |  |  |  |  | 6 | |  |  | | 4 | |  | | 4 | | 8 | |  | |  | |  | |  | |  | |  | |  | |  | |  | |  | | |  | |  | | 22 | |
| *Artemisia lactiflora* | 3 |  |  |  | 5 | |  |  | |  | |  | |  | |  | |  | |  | |  | |  | |  | |  | |  | |  | |  | |  | | |  | |  | | 8 | |
| *Cassia leschenaultiana DC.* |  |  |  |  |  | |  | 1 | |  | |  | | 6 | |  | |  | |  | |  | |  | |  | |  | |  | |  | |  | |  | | |  | |  | | 7 | |
| *Pinellia ternata* |  |  |  |  |  | |  |  | |  | |  | | 7 | |  | |  | |  | |  | |  | |  | |  | |  | |  | |  | |  | | |  | |  | | 7 | |
| *Imperata cylindrica* |  |  |  |  | 7 | |  |  | |  | |  | |  | |  | |  | |  | |  | |  | |  | |  | |  | |  | |  | |  | | |  | |  | | 7 | |
| *Chenopodium album L.* |  |  |  | 6 |  | |  |  | |  | |  | |  | |  | |  | |  | |  | |  | |  | |  | |  | |  | |  | |  | | |  | |  | | 6 | |
| *Fagopyrum dibotrys (D. Don) Hara* |  |  |  |  |  | |  |  | |  | |  | |  | | 4 | |  | |  | |  | |  | |  | |  | |  | |  | |  | |  | | |  | |  | | 4 | |
| *Commelina communis L.* |  |  |  | 3 |  | |  |  | |  | |  | |  | |  | |  | |  | |  | |  | |  | |  | |  | |  | |  | |  | | |  | |  | | 3 | |
| *Atropa belladonna L.* |  |  |  |  |  | |  |  | |  | |  | | 3 | |  | |  | |  | |  | |  | |  | |  | |  | |  | |  | |  | | |  | |  | | 3 | |
| *Geranium wilfordii Maxim.* |  |  |  |  |  | |  |  | |  | |  | |  | |  | |  | |  | |  | |  | | 3 | |  | |  | |  | |  | |  | | |  | |  | | 3 | |
| *Rubus innominatus S. Moore* |  |  |  |  |  | |  |  | |  | |  | | 2 | |  | |  | |  | |  | |  | |  | |  | |  | |  | |  | |  | | |  | |  | | 2 | |
| *Datura stramonium Linn.* |  |  |  |  |  | |  |  | |  | |  | | 2 | |  | |  | |  | |  | |  | |  | |  | |  | |  | |  | |  | | |  | |  | | 2 | |
| *Daucus carota L.* |  |  |  |  |  | |  |  | | 2 | |  | |  | |  | |  | |  | |  | |  | |  | |  | |  | |  | |  | |  | | |  | |  | | 2 | |
| *Acorus calamus L.* | 1 |  |  |  |  | |  |  | |  | |  | |  | |  | |  | |  | |  | |  | |  | |  | |  | |  | |  | |  | | |  | |  | | 1 | |
| *Rorippa indica (L.)Hiern* |  |  |  |  |  | |  |  | |  | |  | |  | |  | |  | |  | |  | |  | |  | | 1 | |  | |  | |  | |  | | |  | |  | | 1 | |
| **VINE** |  |  |  |  |  | |  |  | |  | |  | |  | |  | |  | |  | |  | |  | |  | |  | |  | |  | |  | |  | | |  | |  | |  | |
| *Ficus tikoua* |  |  |  |  |  | |  | 5 | | 4 | |  | |  | |  | |  | |  | |  | |  | | 7 | | 1 | |  | |  | | 5 | |  | | | 7 | |  | | 29 | |
| *Smilax china  L.* |  |  |  |  |  | |  |  | |  | |  | |  | |  | |  | |  | |  | |  | |  | |  | |  | | 6 | |  | |  | | |  | | 6 | | 12 | |
| *Holboellia latifolia Wall* |  |  |  |  |  | |  |  | |  | |  | |  | |  | |  | |  | |  | |  | |  | |  | |  | | 4 | |  | |  | | |  | |  | | 4 | |
| **TREE** |  |  |  |  |  | |  |  | |  | |  | |  | |  | |  | |  | |  | |  | |  | |  | |  | |  | |  | |  | | |  | |  | |  | |
| *Zanthoxylum planispinum Sieb.et Zucc.* |  | 1 |  |  |  | |  | 6 | |  | |  | | 4 | |  | |  | | 7 | | 6 | |  | | 4 | | 3 | |  | | 5 | | 5 | |  | | | 3 | | 15 | | 59 | |
| *Myrsine africana Linn.* |  |  |  |  |  | |  |  | |  | |  | |  | |  | |  | |  | | 3 | |  | |  | | 2 | |  | | 5 | | 8 | |  | | | 6 | | 7 | | 31 | |
| *Indigofera pseudotinctoria* |  |  |  |  |  | |  | 3 | |  | |  | | 3 | |  | |  | | 2 | |  | |  | |  | |  | |  | |  | |  | |  | | | 4 | | 6 | | 18 | |
| *Phyllostachys pubescens Mazel ex H. de Lehaie* |  |  |  |  |  | |  |  | |  | |  | |  | |  | |  | |  | |  | |  | |  | |  | |  | |  | | 3 | |  | | | 2 | | 6 | | 11 | |
| *Litsea rubescens* | |  |  |  |  | |  |  | |  | |  | | 2 | |  | |  | | 2 | |  | |  | |  | |  | |  | |  | |  | |  | | | 2 | |  | | 6 | |
| *Rubus pinfaensis* |  |  |  |  |  | |  |  | | 3 | |  | |  | |  | |  | |  | |  | |  | |  | |  | |  | |  | |  | |  | | | 2 | |  | | 5 | |
| *Hypericum patulum* |  |  |  |  |  | |  |  | |  | |  | |  | |  | |  | |  | |  | |  | |  | |  | |  | | 3 | | 2 | |  | | |  | |  | | 5 | |
| *Quercus fabri* |  |  |  |  |  | |  |  | |  | |  | |  | |  | |  | |  | |  | |  | |  | |  | |  | | 5 | |  | |  | | |  | |  | | 5 | |
|  |  |  |  |  |  | |  |  | |  | |  | |  | |  | |  | |  | |  | |  | |  | |  | |  | |  | |  | |  | | |  | |  | |  | |
| *Carpinus pubescens* | |  |  |  |  | |  |  | |  | |  | |  | |  | |  | |  | |  | |  | |  | |  | |  | |  | | 3 | |  | | | 6 | | 4 | | 13 | |
| *Ilex chinensis* |  |  |  |  |  | |  |  | |  | |  | |  | |  | |  | |  | |  | |  | |  | |  | |  | |  | | 2 | |  | | | 2 | |  | | 4 | |
| *Celtis sinensis Pers.*   \| *Broussonetia papyrifera* \| \| --- \| |  |  |  |  |  | |  |  | |  | |  | |  | |  | |  | |  | |  | |  | |  | |  | |  | |  | |  | |  | | |  | | 2 | | 2 | |
|  |  |  |  |  |  | |  |  | |  | |  | | 2 | |  | |  | | 1 | |  | |  | |  | |  | |  | |  | |  | |  | | |  | |  | | 4 | |
| *Itea yunnanensis* |  |  |  |  |  | |  | 3 | |  | |  | | 1 | |  | |  | | 3 | |  | |  | | 1 | |  | |  | | 9 | |  | |  | | | 4 | | 2 | | 23 | |
| Total | 491 | 302 |  | 751 | 328 | |  | 621 | | 292 | |  | | 685 | | 306 | |  | | 590 | | 225 | |  | | 504 | | 259 | |  | | 868 | | 469 | |  | | | 528 | | 480 | | 7699 | |
