# Supplementary material for: A study of soil seed banks across one complete chronosequence of secondary succession in a karst landscape
Source: PeerJ. 2020 Oct 19;8:e10226. doi: 10.7717/peerj.10226 (PMC7580579; doi:10.7717/peerj.10226)
Supplement: Supplemental Information 4 [file peerj-08-10226-s004.docx]

Supplemental Information III:

1. **One-way ANOVA on seed density in seed banks before seed germination at eight succession**

| **Descriptive statistics**   \| Seed density (number. m^-2^) \| \| \| \| \| \| \| \| \| \| \| --- \| --- \| --- \| --- \| --- \| --- \| --- \| --- \| --- \| --- \| \| Succession stages \| N \| Mean \| Standard Deviation \| Standard Error \| The 95% confidence interval of mean \| \| Min. \| Max \| 元件間變異數 \| \| Lower limit \| Upper limit \| \| GC-I \| 60 \| .8629 \| .26367 \| .03404 \| .7947 \| .9310 \| .37 \| 1.53 \|  \| \| GC-II \| 60 \| .9651 \| .24568 \| .03172 \| .9016 \| 1.0286 \| .37 \| 1.48 \|  \| \| SGC-I \| 60 \| .8715 \| .20776 \| .02682 \| .8178 \| .9251 \| .37 \| 1.32 \|  \| \| SGC-II \| 60 \| .8827 \| .26362 \| .03403 \| .8146 \| .9508 \| .37 \| 1.43 \|  \| \| SF \| 60 \| .7430 \| .24226 \| .03128 \| .6804 \| .8056 \| .37 \| 1.23 \|  \| \| TVSF \| 60 \| .6884 \| .26972 \| .03482 \| .6187 \| .7581 \| .00 \| 1.06 \|  \| \| SEBF \| 60 \| .8491 \| .18524 \| .02391 \| .8012 \| .8969 \| .34 \| 1.10 \|  \| \| PEBF \| 60 \| .9043 \| .17679 \| .02552 \| .8530 \| .9557 \| .34 \| 1.11 \|  \| \| Total \| 480 \| .8444 \| .24849 \| .01149 \| .8218 \| .8669 \| .01 \| 1.53 \|  \|  \| **Homogeneity test of variance** \| \| \| \| \| --- \| --- \| --- \| --- \| \| Seed density (number. m^-2^) \| \| \| \| \| Levene statistical quantity \| df1 \| df2 \| Significance \| \| 2.017 \| 7 \| 472 \| 0.052 \|  \| **ANOVA** \| \| \| \| \| \| \| --- \| --- \| --- \| --- \| --- \| --- \| \| Seed density (number. m^-2^) \| \| \| \| \| \| \|  \| Sum of squares \| df \| Mean square \| F \| Significance \| \| Between groups \| 3.278 \| 7 \| .468 \| 8.428 \| ＜0.001 \| \| Within group \| 25.558 \| 472 \| .056 \|  \|  \| \| Total \| 28.836 \| 479 \|  \|  \|  \| |
| --- | --- | --- | --- | --- | --- | --- | --- | --- | --- | --- | --- | --- | --- | --- | --- | --- | --- | --- | --- | --- | --- | --- | --- | --- | --- | --- | --- | --- | --- | --- | --- | --- | --- | --- | --- | --- | --- | --- | --- | --- | --- | --- | --- | --- | --- | --- | --- | --- | --- | --- | --- | --- | --- | --- | --- | --- | --- | --- | --- | --- | --- | --- | --- | --- | --- | --- | --- | --- | --- | --- | --- | --- | --- | --- | --- | --- | --- | --- | --- | --- | --- | --- | --- | --- | --- | --- | --- | --- | --- | --- | --- | --- | --- | --- | --- | --- | --- | --- | --- | --- | --- | --- | --- | --- | --- | --- | --- | --- | --- | --- | --- | --- | --- | --- | --- | --- | --- | --- | --- | --- | --- | --- | --- | --- | --- | --- | --- | --- | --- | --- | --- | --- | --- | --- | --- | --- | --- | --- | --- | --- | --- | --- | --- | --- | --- | --- | --- | --- | --- | --- | --- | --- | --- | --- | --- | --- | --- | --- | --- | --- | --- | --- | --- | --- |

| **Multiple comparisons** | | | | | | | |
| --- | --- | --- | --- | --- | --- | --- | --- |
| Seed density (number. m^-2^)  LSD | | | | | | | |
| (I) group | | (J) group | Mean difference  (I-J) | Standard Error | Significance | 95% confidence interval | |
|  |  |  |  |  |  | Lower limit | Upper limit |
| LSD | GC-I | GC-II | -.10226^*^ | .04304 | .018 | -.1868 | -.0177 |
|  |  | SGC-I | -.00862 | .04304 | .841 | -.0932 | .0759 |
|  |  | SGC-II | -.01985 | .04304 | .645 | -.1044 | .0647 |
|  |  | SF | .11987^*^ | .04304 | .006 | .0353 | .2044 |
|  |  | TVSF | .17447^*^ | .04304 | ＜0.001 | .0899 | .2590 |
|  |  | SEBF | .01376 | .04304 | .749 | -.0708 | .0983 |
|  |  | PEBF | -.04149 | .04565 | .364 | -.1312 | .0482 |
|  | GC-II | GC-I | .10226^*^ | .04304 | .018 | .0177 | .1868 |
|  |  | SGC-I | .09363^*^ | .04304 | .030 | .0091 | .1782 |
|  |  | SGC-II | .08240 | .04304 | .056 | -.0022 | .1670 |
|  |  | TVSF | .22213^*^ | .04304 | ＜0.001 | .1376 | .3067 |
|  |  | SF | .27672^*^ | .04304 | ＜0.001 | .1922 | .3613 |
|  |  | SEBF | .11601^*^ | .04304 | .007 | .0314 | .2006 |
|  |  | PEBF | .06077 | .04565 | .184 | -.0289 | .1505 |
|  | SGC-I | GC-I | .00862 | .04304 | .841 | -.0759 | .0932 |
|  |  | GC-II | -.09363^*^ | .04304 | .030 | -.1782 | -.0091 |
|  |  | SGC-II | -.01123 | .04304 | .794 | -.0958 | .0733 |
|  |  | TVSF | .12849^*^ | .04304 | .003 | .0439 | .2131 |
|  |  | SF | .18309^*^ | .04304 | ＜0.001 | .0985 | .2677 |
|  |  | SEBF | .02238 | .04304 | .603 | -.0622 | .1070 |
|  |  | PEBF | -.03287 | .04565 | .472 | -.1226 | .0568 |
|  | SGC-II | GC-I | .01985 | .04304 | .645 | -.0647 | .1044 |
|  |  | GC-II | -.08240 | .04304 | .056 | -.1670 | .0022 |
|  |  | SGC-I | .01123 | .04304 | .794 | -.0733 | .0958 |
|  |  | TVSF | .13972^*^ | .04304 | .001 | .0552 | .2243 |
|  |  | SF | .19432^*^ | .04304 | ＜0.001 | .1098 | .2789 |
|  |  | SEBF | .03361 | .04304 | .435 | -.0510 | .1182 |
|  |  | PEBF | -.02164 | .04565 | .636 | -.1113 | .0681 |
|  | TVSF | GC-I | -.11987^*^ | .04304 | .006 | -.2044 | -.0353 |
|  |  | GC-II | -.22213^*^ | .04304 | ＜0.001 | -.3067 | -.1376 |
|  |  | SGC-I | -.12849^*^ | .04304 | .003 | -.2131 | -.0439 |
|  |  | SGC-II | -.13972^*^ | .04304 | .001 | -.2243 | -.0552 |
|  |  | SF | .05460 | .04304 | .205 | -.0300 | .1392 |
|  |  | SEBF | -.10611^*^ | .04304 | .014 | -.1907 | -.0215 |
|  |  | PEBF | -.16136^*^ | .04565 | ＜0.001 | -.2511 | -.0717 |
|  | SF | GC-I | -.17447^*^ | .04304 | ＜0.001 | -.2590 | -.0899 |
|  |  | GC-II | -.27672^*^ | .04304 | ＜0.001 | -.3613 | -.1922 |
|  |  | SGC-I | -.18309^*^ | .04304 | ＜0.001 | -.2677 | -.0985 |
|  |  | SGC-II | -.19432^*^ | .04304 | ＜0.001 | -.2789 | -.1098 |
|  |  | TVSF | -.05460 | .04304 | .205 | -.1392 | .0300 |
|  |  | SEBF | -.16071^*^ | .04304 | ＜0.001 | -.2453 | -.0761 |
|  |  | PEBF | -.21596^*^ | .04565 | ＜0.001 | -.3057 | -.1263 |
|  | SEBF | GC-I | -.01376 | .04304 | .749 | -.0983 | .0708 |
|  |  | GC-II | -.11601^*^ | .04304 | .007 | -.2006 | -.0314 |
|  |  | SGC-I | -.02238 | .04304 | .603 | -.1070 | .0622 |
|  |  | SGC-II | -.03361 | .04304 | .435 | -.1182 | .0510 |
|  |  | TVSF | .10611^*^ | .04304 | .014 | .0215 | .1907 |
|  |  | SF | .16071^*^ | .04304 | ＜0.001 | .0761 | .2453 |
|  |  | PEBF | -.05525 | .04565 | .227 | -.1449 | .0345 |
|  | PEBF | GC-I | .04149 | .04565 | .364 | -.0482 | .1312 |
|  |  | GC-II | -.06077 | .04565 | .184 | -.1505 | .0289 |
|  |  | SGC-I | .03287 | .04565 | .472 | -.0568 | .1226 |
|  |  | SGC-II | .02164 | .04565 | .636 | -.0681 | .1113 |
|  |  | TVSF | .16136^*^ | .04565 | ＜0.001 | .0717 | .2511 |
|  |  | SF | .21596^*^ | .04565 | ＜0.001 | .1263 | .3057 |
|  |  | SEBF | .05525 | .04565 | .227 | -.0345 | .1449 |
| *. The significance level of mean difference was 0.05 | | | | | | | |

1. **One-way ANOVA on seed density in seed banks after seed germination at eight succession**

**Descriptive statistics**

Seed density (number. m^-2^)

| Succession stage | N | Mean | Standard Deviation | Standard Error | The 95% confidence interval of mean | | Min. | Max | 元件間變異數 |
| --- | --- | --- | --- | --- | --- | --- | --- | --- | --- |
|  |  |  |  |  | Lower limit | Upper limit |  |  |  |
| GC-I | 60 | .8884 | .21521 | .02778 | .8328 | .9439 | .65 | 1.32 |  |
| GC-II | 60 | .8790 | .23019 | .02972 | .8195 | .9384 | .65 | 1.54 |  |
| SGC-I | 60 | .8247 | .18830 | .02431 | .7761 | .8733 | .65 | 1.49 |  |
| SGC-II | 60 | .9375 | .19918 | .02571 | .8861 | .9890 | .65 | 1.32 |  |
| SF | 60 | .8814 | .19649 | .02537 | .8306 | .9321 | .65 | 1.24 |  |
| TVSF | 60 | .9586 | .18469 | .02384 | .9109 | 1.0064 | .65 | 1.38 |  |
| SEBF | 60 | .8113 | .22854 | .02950 | .7522 | .8703 | .30 | 1.07 |  |
| PEBF | 60 | .8150 | .16448 | .02374 | .7673 | .8628 | .30 | 1.12 |  |
| Total | 480 | .8760 | .20783 | .00961 | .8571 | .8949 | .30 | 1.54 |  |

| **Homogeneity test of variance** | | | |
| --- | --- | --- | --- |
| Seed density (number. m^-2^) | | | |
| Levene statistical quantity | df1 | df2 | Significance |
| 1.692 | 7 | 472 | .109 |

| **ANOVA** | | | | | |
| --- | --- | --- | --- | --- | --- |
| Sed density (number.m^-2^) | | | | | |
|  | Sum of square | df | Mean variance | F | Significance |
| Between groups | 1.236 | 7 | .177 | 4.290 | ＜0.001 |
| Within group | 18.935 | 472 | .041 |  |  |
| Total | 20.171 | 479 |  |  |  |

| Multiple comparison | | | | | | | |
| --- | --- | --- | --- | --- | --- | --- | --- |
| Sed density (number.m^-2^)  LSD | | | | | | | |
| (I) group | | (J) group | Mean difference (I-J) | Standard error | Significance | 95% confidence interval | |
|  |  |  |  |  |  | Lower limit | Lower limit |
| LSD | GC-I | GC-II | .00940 | .03704 | .800 | -.0634 | .0822 |
|  |  | SGC-I | .06365 | .03704 | .086 | -.0091 | .1364 |
|  |  | SGC-II | -.04919 | .03704 | .185 | -.1220 | .0236 |
|  |  | SF | .00698 | .03704 | .851 | -.0658 | .0798 |
|  |  | TVSF | -.07029 | .03704 | .058 | -.1431 | .0025 |
|  |  | SEBF | .07707^*^ | .03704 | .038 | .0043 | .1499 |
|  |  | PEBF | .07332 | .03929 | .063 | -.0039 | .1505 |
|  | GC-II | GC-I | -.00940 | .03704 | .800 | -.0822 | .0634 |
|  |  | SGC-I | .05425 | .03704 | .144 | -.0185 | .1270 |
|  |  | SGC-II | -.05859 | .03704 | .114 | -.1314 | .0142 |
|  |  | TVSF | -.00242 | .03704 | .948 | -.0752 | .0704 |
|  |  | SF | -.07969^*^ | .03704 | .032 | -.1525 | -.0069 |
|  |  | SEBF | .06767 | .03704 | .068 | -.0051 | .1405 |
|  |  | PEBF | .06392 | .03929 | .104 | -.0133 | .1411 |
|  | SGC-I | GC-I | -.06365 | .03704 | .086 | -.1364 | .0091 |
|  |  | GC-II | -.05425 | .03704 | .144 | -.1270 | .0185 |
|  |  | SGC-II | -.11284^*^ | .03704 | .002 | -.1856 | -.0400 |
|  |  | TVSF | -.05667 | .03704 | .127 | -.1295 | .0161 |
|  |  | SF | -.13394^*^ | .03704 | ＜0.001 | -.2067 | -.0611 |
|  |  | SEBF | .01342 | .03704 | .717 | -.0594 | .0862 |
|  |  | PEBF | .00967 | .03929 | .806 | -.0675 | .0869 |
|  | SGC-II | GC-I | .04919 | .03704 | .185 | -.0236 | .1220 |
|  |  | GC-II | .05859 | .03704 | .114 | -.0142 | .1314 |
|  |  | SGC-I | .11284^*^ | .03704 | .002 | .0400 | .1856 |
|  |  | TVSF | .05617 | .03704 | .130 | -.0166 | .1290 |
|  |  | SF | -.02110 | .03704 | .569 | -.0939 | .0517 |
|  |  | SEBF | .12626^*^ | .03704 | .001 | .0535 | .1990 |
|  |  | PEBF | .12251^*^ | .03929 | .002 | .0453 | .1997 |
|  | TVSF | GC-I | -.00698 | .03704 | .851 | -.0798 | .0658 |
|  |  | GC-II | .00242 | .03704 | .948 | -.0704 | .0752 |
|  |  | SGC-I | .05667 | .03704 | .127 | -.0161 | .1295 |
|  |  | SGC-II | -.05617 | .03704 | .130 | -.1290 | .0166 |
|  |  | SF | -.07727^*^ | .03704 | .038 | -.1501 | -.0045 |
|  |  | SEBF | .07008 | .03704 | .059 | -.0027 | .1429 |
|  |  | PEBF | .06634 | .03929 | .092 | -.0109 | .1435 |
|  | SF | GC-I | .07029 | .03704 | .058 | -.0025 | .1431 |
|  |  | GC-II | .07969^*^ | .03704 | .032 | .0069 | .1525 |
|  |  | SGC-I | .13394^*^ | .03704 | ＜0.001 | .0611 | .2067 |
|  |  | SGC-II | .02110 | .03704 | .569 | -.0517 | .0939 |
|  |  | TVSF | .07727^*^ | .03704 | .038 | .0045 | .1501 |
|  |  | SEBF | .14735^*^ | .03704 | ＜0.001 | .0746 | .2201 |
|  |  | PEBF | .14361^*^ | .03929 | ＜0.001 | .0664 | .2208 |
|  | SEBF | GC-I | -.07707^*^ | .03704 | .038 | -.1499 | -.0043 |
|  |  | GC-II | -.06767 | .03704 | .068 | -.1405 | .0051 |
|  |  | SGC-I | -.01342 | .03704 | .717 | -.0862 | .0594 |
|  |  | SGC-II | -.12626^*^ | .03704 | .001 | -.1990 | -.0535 |
|  |  | TVSF | -.07008 | .03704 | .059 | -.1429 | .0027 |
|  |  | SF | -.14735^*^ | .03704 | ＜0.001 | -.2201 | -.0746 |
|  |  | PEBF | -.00374 | .03929 | .924 | -.0810 | .0735 |
|  | PEBF | GC-I | -.07332 | .03929 | .063 | -.1505 | .0039 |
|  |  | GC-II | -.06392 | .03929 | .104 | -.1411 | .0133 |
|  |  | SGC-I | -.00967 | .03929 | .806 | -.0869 | .0675 |
|  |  | SGC-II | -.12251^*^ | .03929 | .002 | -.1997 | -.0453 |
|  |  | TVSF | -.06634 | .03929 | .092 | -.1435 | .0109 |
|  |  | SF | -.14361^*^ | .03929 | ＜0.001 | -.2208 | -.0664 |
|  |  | SEBF | .00374 | .03929 | .924 | -.0735 | .0810 |
| *. The significance level of mean difference was 0.05 | | | | | | | |

1. **One-way ANOVA on seed density in seed banks between before and after seed germination at eight succession stages.** In the following table, 1.0-8.0 represents the succession stages before seed germination; 9.0-18.0 represents the succession stages after seed germination

Descriptive statistics

| Send density (number.m^-2^) | | | | | | | | | |
| --- | --- | --- | --- | --- | --- | --- | --- | --- | --- |
| Succession stage | N | Mean | Standard deviation | Standard error | The 95% confidence interval of mean | | Min. | Max. | 元件間變異數 |
|  |  |  |  |  | Lower limit | Upper limit |  |  |  |
| 1.00 | 60 | .8629 | .26367 | .03404 | .7947 | .9310 | .37 | 1.53 |  |
| 2.00 | 60 | .9651 | .24568 | .03172 | .9016 | 1.0286 | .37 | 1.48 |  |
| 3.00 | 60 | .8715 | .20776 | .02682 | .8178 | .9251 | .37 | 1.32 |  |
| 4.00 | 60 | .8827 | .26362 | .03403 | .8146 | .9508 | .37 | 1.43 |  |
| 5.00 | 60 | .7430 | .24226 | .03128 | .6804 | .8056 | .37 | 1.23 |  |
| 6.00 | 60 | .6884 | .26972 | .03482 | .6187 | .7581 | .00 | 1.06 |  |
| 7.00 | 60 | .8491 | .18524 | .02391 | .8012 | .8969 | .34 | 1.10 |  |
| 8.00 | 60 | .9043 | .17679 | .02552 | .8530 | .9557 | .34 | 1.11 |  |
| 9.00 | 60 | .8884 | .21521 | .02778 | .8328 | .9439 | .65 | 1.32 |  |
| 10.00 | 60 | .8790 | .23019 | .02972 | .8195 | .9384 | .65 | 1.54 |  |
| 11.00 | 60 | .8247 | .18830 | .02431 | .7761 | .8733 | .65 | 1.49 |  |
| 12.00 | 60 | .9375 | .19918 | .02571 | .8861 | .9890 | .65 | 1.32 |  |
| 13.00 | 60 | .8814 | .19649 | .02537 | .8306 | .9321 | .65 | 1.24 |  |
| 14.00 | 60 | .9586 | .18469 | .02384 | .9109 | 1.0064 | .65 | 1.38 |  |
| 15.00 | 60 | .8113 | .22854 | .02950 | .7522 | .8703 | .30 | 1.07 |  |
| 16.00 | 60 | .8278 | .16004 | .02066 | .7864 | .8691 | .30 | 1.12 |  |
| Total | 960 | .8604 | .22850 | .00742 | .8459 | .8750 | .00 | 1.54 |  |

| **Homogeneity test of variance** | | | |
| --- | --- | --- | --- |
| Seed density (number m^-2^) | | | |
| Sum of square | df | Mean variable | F |
| 2.183 | 15 | 944 | .06 |

| **ANOVA** | | | | | |
| --- | --- | --- | --- | --- | --- |
|  | Sum of square | df | Mean variable | F | Significance |
| Between groups | 4.713 | 15 | .314 | 6.547 | ＜0.001 |
| Within group | 44.733 | 944 | .048 |  |  |
| Total | 49.447 | 959 |  |  |  |

| **Multiple comparison** | | | | | | | |
| --- | --- | --- | --- | --- | --- | --- | --- |
| Seed density  LSD | | | | | | | |
| (I) group | | (J) group | Mean difference (I-J) | Standard error | Significance | 95% confidence interval | |
|  |  |  |  |  |  | Lower limit | Upper limit |
| LSD | 1.00 | 2.00 | -.10226^*^ | .04000 | .011 | -.1808 | -.0238 |
|  |  | 3.00 | -.00862 | .04000 | .829 | -.0871 | .0699 |
|  |  | 4.00 | -.01985 | .04000 | .620 | -.0984 | .0586 |
|  |  | 5.00 | .11987^*^ | .04000 | .003 | .0414 | .1984 |
|  |  | 6.00 | .17447^*^ | .04000 | ＜0.001 | .0960 | .2530 |
|  |  | 7.00 | .01376 | .04000 | .731 | -.0647 | .0923 |
|  |  | 8.00 | -.04149 | .04243 | .328 | -.1248 | .0418 |
|  |  | 9.00 | -.02550 | .04000 | .524 | -.1040 | .0530 |
|  |  | 10.00 | -.01610 | .04000 | .687 | -.0946 | .0624 |
|  |  | 11.00 | .03815 | .04000 | .340 | -.0403 | .1166 |
|  |  | 12.00 | -.07469 | .04000 | .062 | -.1532 | .0038 |
|  |  | 13.00 | -.01852 | .04000 | .644 | -.0970 | .0600 |
|  |  | 14.00 | -.09579^*^ | .04000 | .017 | -.1743 | -.0173 |
|  |  | 15.00 | .05157 | .04000 | .198 | -.0269 | .1301 |
|  |  | 16.00 | .03508 | .04000 | .381 | -.0434 | .1136 |
|  | 2.00 | 1.00 | .10226^*^ | .04000 | .011 | .0238 | .1808 |
|  |  | 3.00 | .09363^*^ | .04000 | .019 | .0151 | .1721 |
|  |  | 4.00 | .08240^*^ | .04000 | .040 | .0039 | .1609 |
|  |  | 5.00 | .22213^*^ | .04000 | ＜0.001 | .1436 | .3006 |
|  |  | 6.00 | .27672^*^ | .04000 | ＜0.001 | .1982 | .3552 |
|  |  | 7.00 | .11601^*^ | .04000 | .004 | .0375 | .1945 |
|  |  | 8.00 | .06077 | .04243 | .152 | -.0225 | .1440 |
|  |  | 9.00 | .07676 | .04000 | .055 | -.0017 | .1553 |
|  |  | 10.00 | .08616^*^ | .04000 | .031 | .0077 | .1647 |
|  |  | 11.00 | .14041^*^ | .04000 | ＜0.001 | .0619 | .2189 |
|  |  | 12.00 | .02757 | .04000 | .491 | -.0509 | .1061 |
|  |  | 13.00 | .08374^*^ | .04000 | .037 | .0052 | .1622 |
|  |  | 14.00 | .00647 | .04000 | .872 | -.0720 | .0850 |
|  |  | 15.00 | .15382^*^ | .04000 | ＜0.001 | .0753 | .2323 |
|  |  | 16.00 | .13733^*^ | .04000 | .001 | .0588 | .2158 |
|  | 3.00 | 1.00 | .00862 | .04000 | .829 | -.0699 | .0871 |
|  |  | 2.00 | -.09363^*^ | .04000 | .019 | -.1721 | -.0151 |
|  |  | 4.00 | -.01123 | .04000 | .779 | -.0897 | .0673 |
|  |  | 5.00 | .12849^*^ | .04000 | .001 | .0500 | .2070 |
|  |  | 6.00 | .18309^*^ | .04000 | ＜0.001 | .1046 | .2616 |
|  |  | 7.00 | .02238 | .04000 | .576 | -.0561 | .1009 |
|  |  | 8.00 | -.03287 | .04243 | .439 | -.1161 | .0504 |
|  |  | 9.00 | -.01688 | .04000 | .673 | -.0954 | .0616 |
|  |  | 10.00 | -.00748 | .04000 | .852 | -.0860 | .0710 |
|  |  | 11.00 | .04677 | .04000 | .243 | -.0317 | .1253 |
|  |  | 12.00 | -.06607 | .04000 | .099 | -.1446 | .0124 |
|  |  | 13.00 | -.00989 | .04000 | .805 | -.0884 | .0686 |
|  |  | 14.00 | -.08716^*^ | .04000 | .030 | -.1657 | -.0087 |
|  |  | 15.00 | .06019 | .04000 | .133 | -.0183 | .1387 |
|  |  | 16.00 | .04370 | .04000 | .275 | -.0348 | .1222 |
|  | 4.00 | 1.00 | .01985 | .04000 | .620 | -.0586 | .0984 |
|  |  | 2.00 | -.08240^*^ | .04000 | .040 | -.1609 | -.0039 |
|  |  | 3.00 | .01123 | .04000 | .779 | -.0673 | .0897 |
|  |  | 5.00 | .13972^*^ | .04000 | ＜0.001 | .0612 | .2182 |
|  |  | 6.00 | .19432^*^ | .04000 | ＜0.001 | .1158 | .2728 |
|  |  | 7.00 | .03361 | .04000 | .401 | -.0449 | .1121 |
|  |  | 8.00 | -.02164 | .04243 | .610 | -.1049 | .0616 |
|  |  | 9.00 | -.00565 | .04000 | .888 | -.0841 | .0729 |
|  |  | 10.00 | .00375 | .04000 | .925 | -.0747 | .0823 |
|  |  | 11.00 | .05800 | .04000 | .147 | -.0205 | .1365 |
|  |  | 12.00 | -.05484 | .04000 | .171 | -.1333 | .0237 |
|  |  | 13.00 | .00134 | .04000 | .973 | -.0772 | .0798 |
|  |  | 14.00 | -.07593 | .04000 | .058 | -.1544 | .0026 |
|  |  | 15.00 | .07142 | .04000 | .074 | -.0071 | .1499 |
|  |  | 16.00 | .05493 | .04000 | .170 | -.0236 | .1334 |
|  | 5.00 | 1.00 | -.11987^*^ | .04000 | .003 | -.1984 | -.0414 |
|  |  | 2.00 | -.22213^*^ | .04000 | ＜0.001 | -.3006 | -.1436 |
|  |  | 3.00 | -.12849^*^ | .04000 | .001 | -.2070 | -.0500 |
|  |  | 4.00 | -.13972^*^ | .04000 | ＜0.001 | -.2182 | -.0612 |
|  |  | 6.00 | .05460 | .04000 | .173 | -.0239 | .1331 |
|  |  | 7.00 | -.10611^*^ | .04000 | .008 | -.1846 | -.0276 |
|  |  | 8.00 | -.16136^*^ | .04243 | ＜0.001 | -.2446 | -.0781 |
|  |  | 9.00 | -.14537^*^ | .04000 | ＜0.001 | -.2239 | -.0669 |
|  |  | 10.00 | -.13597^*^ | .04000 | .001 | -.2145 | -.0575 |
|  |  | 11.00 | -.08172^*^ | .04000 | .041 | -.1602 | -.0032 |
|  |  | 12.00 | -.19456^*^ | .04000 | ＜0.001 | -.2731 | -.1161 |
|  |  | 13.00 | -.13839^*^ | .04000 | .001 | -.2169 | -.0599 |
|  |  | 14.00 | -.21566^*^ | .04000 | ＜0.001 | -.2942 | -.1372 |
|  |  | 15.00 | -.06830 | .04000 | .088 | -.1468 | .0102 |
|  |  | 16.00 | -.08479^*^ | .04000 | .034 | -.1633 | -.0063 |
|  | 6.00 | 1.00 | -.17447^*^ | .04000 | ＜0.001 | -.2530 | -.0960 |
|  |  | 2.00 | -.27672^*^ | .04000 | ＜0.001 | -.3552 | -.1982 |
|  |  | 3.00 | -.18309^*^ | .04000 | ＜0.001 | -.2616 | -.1046 |
|  |  | 4.00 | -.19432^*^ | .04000 | ＜0.001 | -.2728 | -.1158 |
|  |  | 5.00 | -.05460 | .04000 | .173 | -.1331 | .0239 |
|  |  | 7.00 | -.16071^*^ | .04000 | ＜0.001 | -.2392 | -.0822 |
|  |  | 8.00 | -.21596^*^ | .04243 | ＜0.001 | -.2992 | -.1327 |
|  |  | 9.00 | -.19997^*^ | .04000 | ＜0.001 | -.2785 | -.1215 |
|  |  | 10.00 | -.19057^*^ | .04000 | ＜0.001 | -.2691 | -.1121 |
|  |  | 11.00 | -.13632^*^ | .04000 | .001 | -.2148 | -.0578 |
|  |  | 12.00 | -.24916^*^ | .04000 | ＜0.001 | -.3277 | -.1707 |
|  |  | 13.00 | -.19298^*^ | .04000 | ＜0.001 | -.2715 | -.1145 |
|  |  | 14.00 | -.27025^*^ | .04000 | ＜0.001 | -.3488 | -.1918 |
|  |  | 15.00 | -.12290^*^ | .04000 | .002 | -.2014 | -.0444 |
|  |  | 16.00 | -.13939^*^ | .04000 | .001 | -.2179 | -.0609 |
|  | 7.00 | 1.00 | -.01376 | .04000 | .731 | -.0923 | .0647 |
|  |  | 2.00 | -.11601^*^ | .04000 | .004 | -.1945 | -.0375 |
|  |  | 3.00 | -.02238 | .04000 | .576 | -.1009 | .0561 |
|  |  | 4.00 | -.03361 | .04000 | .401 | -.1121 | .0449 |
|  |  | 5.00 | .10611^*^ | .04000 | .008 | .0276 | .1846 |
|  |  | 6.00 | .16071^*^ | .04000 | ＜0.001 | .0822 | .2392 |
|  |  | 8.00 | -.05525 | .04243 | .193 | -.1385 | .0280 |
|  |  | 9.00 | -.03926 | .04000 | .327 | -.1178 | .0392 |
|  |  | 10.00 | -.02986 | .04000 | .456 | -.1084 | .0486 |
|  |  | 11.00 | .02439 | .04000 | .542 | -.0541 | .1029 |
|  |  | 12.00 | -.08845^*^ | .04000 | .027 | -.1669 | -.0099 |
|  |  | 13.00 | -.03227 | .04000 | .420 | -.1108 | .0462 |
|  |  | 14.00 | -.10954^*^ | .04000 | .006 | -.1880 | -.0310 |
|  |  | 15.00 | .03781 | .04000 | .345 | -.0407 | .1163 |
|  |  | 16.00 | .02132 | .04000 | .594 | -.0572 | .0998 |
|  | 8.00 | 1.00 | .04149 | .04243 | .328 | -.0418 | .1248 |
|  |  | 2.00 | -.06077 | .04243 | .152 | -.1440 | .0225 |
|  |  | 3.00 | .03287 | .04243 | .439 | -.0504 | .1161 |
|  |  | 4.00 | .02164 | .04243 | .610 | -.0616 | .1049 |
|  |  | 5.00 | .16136^*^ | .04243 | ＜0.001 | .0781 | .2446 |
|  |  | 6.00 | .21596^*^ | .04243 | ＜0.001 | .1327 | .2992 |
|  |  | 7.00 | .05525 | .04243 | .193 | -.0280 | .1385 |
|  |  | 9.00 | .01599 | .04243 | .706 | -.0673 | .0993 |
|  |  | 10.00 | .02539 | .04243 | .550 | -.0579 | .1087 |
|  |  | 11.00 | .07964 | .04243 | .061 | -.0036 | .1629 |
|  |  | 12.00 | -.03320 | .04243 | .434 | -.1165 | .0501 |
|  |  | 13.00 | .02297 | .04243 | .588 | -.0603 | .1062 |
|  |  | 14.00 | -.05430 | .04243 | .201 | -.1376 | .0290 |
|  |  | 15.00 | .09306^*^ | .04243 | .029 | .0098 | .1763 |
|  |  | 16.00 | .07657 | .04243 | .071 | -.0067 | .1598 |
|  | 9.00 | 1.00 | .02550 | .04000 | .524 | -.0530 | .1040 |
|  |  | 2.00 | -.07676 | .04000 | .055 | -.1553 | .0017 |
|  |  | 3.00 | .01688 | .04000 | .673 | -.0616 | .0954 |
|  |  | 4.00 | .00565 | .04000 | .888 | -.0729 | .0841 |
|  |  | 5.00 | .14537^*^ | .04000 | ＜0.001 | .0669 | .2239 |
|  |  | 6.00 | .19997^*^ | .04000 | ＜0.001 | .1215 | .2785 |
|  |  | 7.00 | .03926 | .04000 | .327 | -.0392 | .1178 |
|  |  | 8.00 | -.01599 | .04243 | .706 | -.0993 | .0673 |
|  |  | 10.00 | .00940 | .04000 | .814 | -.0691 | .0879 |
|  |  | 11.00 | .06365 | .04000 | .112 | -.0148 | .1421 |
|  |  | 12.00 | -.04919 | .04000 | .219 | -.1277 | .0293 |
|  |  | 13.00 | .00698 | .04000 | .861 | -.0715 | .0855 |
|  |  | 14.00 | -.07029 | .04000 | .079 | -.1488 | .0082 |
|  |  | 15.00 | .07707 | .04000 | .054 | -.0014 | .1556 |
|  |  | 16.00 | .06058 | .04000 | .130 | -.0179 | .1391 |
|  | 10.00 | 1.00 | .01610 | .04000 | .687 | -.0624 | .0946 |
|  |  | 2.00 | -.08616^*^ | .04000 | .031 | -.1647 | -.0077 |
|  |  | 3.00 | .00748 | .04000 | .852 | -.0710 | .0860 |
|  |  | 4.00 | -.00375 | .04000 | .925 | -.0823 | .0747 |
|  |  | 5.00 | .13597^*^ | .04000 | .001 | .0575 | .2145 |
|  |  | 6.00 | .19057^*^ | .04000 | ＜0.001 | .1121 | .2691 |
|  |  | 7.00 | .02986 | .04000 | .456 | -.0486 | .1084 |
|  |  | 8.00 | -.02539 | .04243 | .550 | -.1087 | .0579 |
|  |  | 9.00 | -.00940 | .04000 | .814 | -.0879 | .0691 |
|  |  | 11.00 | .05425 | .04000 | .175 | -.0242 | .1327 |
|  |  | 12.00 | -.05859 | .04000 | .143 | -.1371 | .0199 |
|  |  | 13.00 | -.00242 | .04000 | .952 | -.0809 | .0761 |
|  |  | 14.00 | -.07969^*^ | .04000 | .047 | -.1582 | -.0012 |
|  |  | 15.00 | .06767 | .04000 | .091 | -.0108 | .1462 |
|  |  | 16.00 | .05118 | .04000 | .201 | -.0273 | .1297 |
|  | 11.00 | 1.00 | -.03815 | .04000 | .340 | -.1166 | .0403 |
|  |  | 2.00 | -.14041^*^ | .04000 | ＜0.001 | -.2189 | -.0619 |
|  |  | 3.00 | -.04677 | .04000 | .243 | -.1253 | .0317 |
|  |  | 4.00 | -.05800 | .04000 | .147 | -.1365 | .0205 |
|  |  | 5.00 | .08172^*^ | .04000 | .041 | .0032 | .1602 |
|  |  | 6.00 | .13632^*^ | .04000 | .001 | .0578 | .2148 |
|  |  | 7.00 | -.02439 | .04000 | .542 | -.1029 | .0541 |
|  |  | 8.00 | -.07964 | .04243 | .061 | -.1629 | .0036 |
|  |  | 9.00 | -.06365 | .04000 | .112 | -.1421 | .0148 |
|  |  | 10.00 | -.05425 | .04000 | .175 | -.1327 | .0242 |
|  |  | 12.00 | -.11284^*^ | .04000 | .005 | -.1913 | -.0343 |
|  |  | 13.00 | -.05667 | .04000 | .157 | -.1352 | .0218 |
|  |  | 14.00 | -.13394^*^ | .04000 | .001 | -.2124 | -.0554 |
|  |  | 15.00 | .01342 | .04000 | .737 | -.0651 | .0919 |
|  |  | 16.00 | -.00307 | .04000 | .939 | -.0816 | .0754 |
|  | 12.00 | 1.00 | .07469 | .04000 | .062 | -.0038 | .1532 |
|  |  | 2.00 | -.02757 | .04000 | .491 | -.1061 | .0509 |
|  |  | 3.00 | .06607 | .04000 | .099 | -.0124 | .1446 |
|  |  | 4.00 | .05484 | .04000 | .171 | -.0237 | .1333 |
|  |  | 5.00 | .19456^*^ | .04000 | ＜0.001 | .1161 | .2731 |
|  |  | 6.00 | .24916^*^ | .04000 | ＜0.001 | .1707 | .3277 |
|  |  | 7.00 | .08845^*^ | .04000 | .027 | .0099 | .1669 |
|  |  | 8.00 | .03320 | .04243 | .434 | -.0501 | .1165 |
|  |  | 9.00 | .04919 | .04000 | .219 | -.0293 | .1277 |
|  |  | 10.00 | .05859 | .04000 | .143 | -.0199 | .1371 |
|  |  | 11.00 | .11284^*^ | .04000 | .005 | .0343 | .1913 |
|  |  | 13.00 | .05617 | .04000 | .161 | -.0223 | .1347 |
|  |  | 14.00 | -.02110 | .04000 | .598 | -.0996 | .0574 |
|  |  | 15.00 | .12626^*^ | .04000 | .002 | .0478 | .2048 |
|  |  | 16.00 | .10977^*^ | .04000 | .006 | .0313 | .1883 |
|  | 13.00 | 1.00 | .01852 | .04000 | .644 | -.0600 | .0970 |
|  |  | 2.00 | -.08374^*^ | .04000 | .037 | -.1622 | -.0052 |
|  |  | 3.00 | .00989 | .04000 | .805 | -.0686 | .0884 |
|  |  | 4.00 | -.00134 | .04000 | .973 | -.0798 | .0772 |
|  |  | 5.00 | .13839^*^ | .04000 | .001 | .0599 | .2169 |
|  |  | 6.00 | .19298^*^ | .04000 | ＜0.001 | .1145 | .2715 |
|  |  | 7.00 | .03227 | .04000 | .420 | -.0462 | .1108 |
|  |  | 8.00 | -.02297 | .04243 | .588 | -.1062 | .0603 |
|  |  | 9.00 | -.00698 | .04000 | .861 | -.0855 | .0715 |
|  |  | 10.00 | .00242 | .04000 | .952 | -.0761 | .0809 |
|  |  | 11.00 | .05667 | .04000 | .157 | -.0218 | .1352 |
|  |  | 12.00 | -.05617 | .04000 | .161 | -.1347 | .0223 |
|  |  | 14.00 | -.07727 | .04000 | .054 | -.1558 | .0012 |
|  |  | 15.00 | .07008 | .04000 | .080 | -.0084 | .1486 |
|  |  | 16.00 | .05359 | .04000 | .181 | -.0249 | .1321 |
|  | 14.00 | 1.00 | .09579^*^ | .04000 | .017 | .0173 | .1743 |
|  |  | 2.00 | -.00647 | .04000 | .872 | -.0850 | .0720 |
|  |  | 3.00 | .08716^*^ | .04000 | .030 | .0087 | .1657 |
|  |  | 4.00 | .07593 | .04000 | .058 | -.0026 | .1544 |
|  |  | 5.00 | .21566^*^ | .04000 | ＜0.001 | .1372 | .2942 |
|  |  | 6.00 | .27025^*^ | .04000 | ＜0.001 | .1918 | .3488 |
|  |  | 7.00 | .10954^*^ | .04000 | .006 | .0310 | .1880 |
|  |  | 8.00 | .05430 | .04243 | .201 | -.0290 | .1376 |
|  |  | 9.00 | .07029 | .04000 | .079 | -.0082 | .1488 |
|  |  | 10.00 | .07969^*^ | .04000 | .047 | .0012 | .1582 |
|  |  | 11.00 | .13394^*^ | .04000 | .001 | .0554 | .2124 |
|  |  | 12.00 | .02110 | .04000 | .598 | -.0574 | .0996 |
|  |  | 13.00 | .07727 | .04000 | .054 | -.0012 | .1558 |
|  |  | 15.00 | .14735^*^ | .04000 | ＜0.001 | .0689 | .2259 |
|  |  | 16.00 | .13086^*^ | .04000 | .001 | .0524 | .2094 |
|  | 15.00 | 1.00 | -.05157 | .04000 | .198 | -.1301 | .0269 |
|  |  | 2.00 | -.15382^*^ | .04000 | ＜0.001 | -.2323 | -.0753 |
|  |  | 3.00 | -.06019 | .04000 | .133 | -.1387 | .0183 |
|  |  | 4.00 | -.07142 | .04000 | .074 | -.1499 | .0071 |
|  |  | 5.00 | .06830 | .04000 | .088 | -.0102 | .1468 |
|  |  | 6.00 | .12290^*^ | .04000 | .002 | .0444 | .2014 |
|  |  | 7.00 | -.03781 | .04000 | .345 | -.1163 | .0407 |
|  |  | 8.00 | -.09306^*^ | .04243 | .029 | -.1763 | -.0098 |
|  |  | 9.00 | -.07707 | .04000 | .054 | -.1556 | .0014 |
|  |  | 10.00 | -.06767 | .04000 | .091 | -.1462 | .0108 |
|  |  | 11.00 | -.01342 | .04000 | .737 | -.0919 | .0651 |
|  |  | 12.00 | -.12626^*^ | .04000 | .002 | -.2048 | -.0478 |
|  |  | 13.00 | -.07008 | .04000 | .080 | -.1486 | .0084 |
|  |  | 14.00 | -.14735^*^ | .04000 | ＜0.001 | -.2259 | -.0689 |
|  |  | 16.00 | -.01649 | .04000 | .680 | -.0950 | .0620 |
|  | 16.00 | 1.00 | -.03508 | .04000 | .381 | -.1136 | .0434 |
|  |  | 2.00 | -.13733^*^ | .04000 | .001 | -.2158 | -.0588 |
|  |  | 3.00 | -.04370 | .04000 | .275 | -.1222 | .0348 |
|  |  | 4.00 | -.05493 | .04000 | .170 | -.1334 | .0236 |
|  |  | 5.00 | .08479^*^ | .04000 | .034 | .0063 | .1633 |
|  |  | 6.00 | .13939^*^ | .04000 | .001 | .0609 | .2179 |
|  |  | 7.00 | -.02132 | .04000 | .594 | -.0998 | .0572 |
|  |  | 8.00 | -.07657 | .04243 | .071 | -.1598 | .0067 |
|  |  | 9.00 | -.06058 | .04000 | .130 | -.1391 | .0179 |
|  |  | 10.00 | -.05118 | .04000 | .201 | -.1297 | .0273 |
|  |  | 11.00 | .00307 | .04000 | .939 | -.0754 | .0816 |
|  |  | 12.00 | -.10977^*^ | .04000 | .006 | -.1883 | -.0313 |
|  |  | 13.00 | -.05359 | .04000 | .181 | -.1321 | .0249 |
|  |  | 14.00 | -.13086^*^ | .04000 | .001 | -.2094 | -.0524 |
|  |  | 15.00 | .01649 | .04000 | .680 | -.0620 | .0950 |
| * The significance level of mean difference was 0.05. | | | | | | | |
